# Administration Dependent Antioxidant Effect of *Carica papaya* Seeds Water Extract

**DOI:** 10.1155/2014/281508

**Published:** 2014-03-25

**Authors:** Elisa Panzarini, Majdi Dwikat, Stefania Mariano, Cristian Vergallo, Luciana Dini

**Affiliations:** Department of Biological and Environmental Science and Technology (Di.S.Te.B.A.), University of Salento, Prov.le Lecce-Monteroni, 73100 Lecce, Italy

## Abstract

*Carica papaya* is widely used in folk medicine as herbal remedy to prevent, protect against, and cure several diseases. These curative properties are based on the presence in different parts of the plant of phytochemical nutrients with antioxidant effect. Seeds are the less exploited part; thus this study is aimed at assessing the antioxidant activities of the *C. papaya* seeds water extract against hydrogen peroxide (H_2_O_2_) oxidative stress in human skin Detroit 550 fibroblasts. *C. papaya* seeds water extract is not toxic and acts as a potent free radical scavenger, providing protection to Detroit 550 fibroblasts that underwent H_2_O_2_ oxidative stress. Data show that (i) the maximum protective effect is achieved by the simultaneous administration of the extract with 1 mM H_2_O_2_; (ii) the extract in presence of an oxidative stress does not increase catalase activity and prevents the release of cytochrome C and the inner mitochondrial transmembrane potential (Δ*ψ*_*m*_) loss; (iii) the extract is more efficient than vitamin C to hamper the oxidative damage; (iv) the purified subfractions of the seeds water extract exert the same antioxidant effect of whole extract. In conclusion, *C. papaya* seeds water extract is potentially useful for protection against oxidative stress.

## 1. Introduction

Oxidative stress, based on imbalance between prooxidants production and antioxidant defences, is involved in aging process and in several human chronic diseases, for example, cancer, atherosclerosis, coronary heart disease, macular degeneration, Alzheimer's disease, inflammation, and emphysema [1]. Vitamins C and E, *β*-carotene, flavonoids, tannins, anthocyanins, and other phenolic compounds are plant derived compounds with antioxidant activities by scavenging free radicals and represent a special group of nutritional supplements. Food rich in these antioxidants plays a key role in the prevention of oxidative stress based diseases [2, 3].

Papaya (*Carica papaya*), a member of the family Caricaceae, is a tropical fruit rich in dietary antioxidants (vitamin C, tocopherols, total phenols, and *β*-carotene) [4] and bioactive phytochemicals with antioxidant activity (benzyl isothiocyanate) [5]. Different parts of* C. papaya* (leaves, barks, roots, latex, fruit, flowers, and seeds) are used in folk medication to treat a broad range of diseases [6]. Several scientific studies validate many of these traditional uses by demonstrating that* C. papaya* displays a wide range of therapeutic activities (i.e., antiprotozoal, antifungal, antibacterial, antiviral, anti-inflammatory, antihypertensive, hypoglycemic and hypolipidemic, wound healing, antitumor, neuroprotective, diuretic, abortifacient, and antifertility) [6, 7]. These properties mainly depend on the antioxidant activity of some secondary metabolites present in the* C. papaya* organs. The studies of the antioxidant nutrients in* C. papaya* have led to the identification of the main compounds that differ in the different organs. Whole fruit extract contains ferulic, p-coumaric, and caffeic acid, carotenoids, and vitamin C that collectively protect human cells from oxidative stresses [4] and promote wound healing and skin repair [8, 9]; leaves extract contains folic acid, vitamins B_12_, A, and C, alkaloids, saponins, glycosides, tannins, and flavonoids [10] with anticancer activity [11, 12] and protection against the alcohol-induced oxidative damage to the gastric mucosa [13]; seeds extract contains different phenolic compounds, vanillic acid, and vitamin C with antioxidant [14–17] and anticancer [5] activities. Thus,* C. papaya* extracts may act as a synergistic therapeutic dietary supplement in patients with oxidative stress related diseases or could be added to formulations to promote wound healing. Even if literature data reporting on papaya fruit protection against H_2_O_2_-induced oxidative damage are conspicuous, up to date, no researches have explored similar antioxidant activity of papaya seeds water extract. Indeed, water extract has the advantage to prevent the use of harmful chemical compounds. It is worth noting to mention that seeds represent the waste of the consumption of papaya fruits, not only as food but also as raw material for medical preparations. Therefore, the aim of this study was to investigate the antioxidant activity of* C. papaya *seeds water extract in human skin Detroit 550 fibroblasts in which the oxidative stress was induced by hydrogen peroxide (H_2_O_2_). Special attention was paid to the modality of administration and to evaluate if the highest antioxidant effect of* C. papaya *seeds water extract depends on the whole crude extract or on specific subfractions.

## 2. Materials and Methods

### 2.1. Chemicals

All chemicals were of analytical grade and provided by Sigma-Aldrich (St. Louis, MO, USA) unless otherwise indicated.

### 2.2. *C. papaya *Seeds Water Extract Preparation and Fractionation

Seeds were removed from mature* C. papaya* fruits, rinsed with water, and left to dry at room temperature (RT). 10 g of seeds were ground in a mortar, and the powder was left in infusion for 24 h (hours) in 500 mL of sterile distilled water at RT. The crude extract was subjected to a short run (800 g) to obtain a pellet representing the seeds wastes. Unless otherwise indicated, fibroblasts were treated for 1, 2, 4, 8, or 24 h with 1 or 2 mg/mL (w/v, in culture medium) of supernatant in the presence or absence of a prooxidant (1 mM H_2_O_2_) with or without a known antioxidant (50, 100, 150, 200, and 250 *μ*M vitamin C).

**Figure 1:**
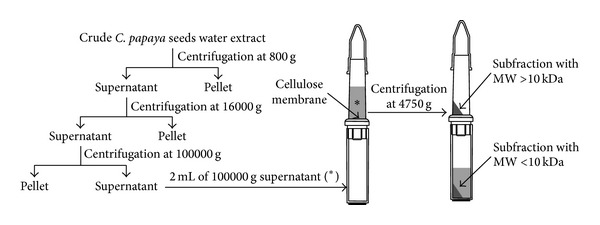
Differential centrifugation of crude* C. papaya *seeds water extract. The crude extract was subjected to a short run at 800 g by Centrifuge 4236 A (Beckman Coulter Inc., Brea, CA, USA) to obtain a first pellet and a supernatant that was further centrifuged at 16000 g to obtain a second pellet and supernatant. The second supernatant was centrifuged at 100000 g to obtain a third pellet and supernatant. This last supernatant was centrifuged at 4750 g by using a Centricon centrifugal filter device with an Ultracel YM-10 regenerated cellulose membrane that permits the ultrafiltration into the lower chamber of substances with molecular weight (MW) < 10 kDa, while substances with MW > 10 kDa are collected in the upper chamber (Merck Millipore, Billerica, MA, USA).

To test also the efficacy of the extract subfractions, the crude whole extract was subjected to short runs by Centrifuge 4236 A (Beckman Coulter Inc., Brea, CA, USA). In particular, the last supernatant obtained at 100000 g was centrifuged at 4750 g by using a Centricon centrifugal filter device with an Ultracel YM-10 (Merck Millipore, Billerica, MA, USA) regenerated cellulose membrane that permits the ultrafiltration into the lower chamber of substances with molecular weight (MW) < 10 kDa, while substances with MW > 10 kDa are collected in the upper chamber. The pellet obtained was added to fibroblasts in order to obtain 1 mg/mL (w/v, in culture medium) final concentration. In Figure 1, the detailed fractioning procedure is illustrated.

### 2.3. Cell Cultures and Treatments

Human skin Detroit 550 fibroblasts were purchased from Centro Substrati Cellulari (Istituto Zooprofilattico Sperimentale, Brescia, Italy) and cultured in 75 cm^2^ flasks (Iwaki, Tokyo, Japan) at a cell density of 10^6^ fibroblasts/mL in Eagle's minimum essential medium (EMEM) with Earle's Balanced Salts (Cambrex BioScience, Verviers, Belgium), supplemented with 10% (v/v) heat-inactivated fetal calf serum (Cambrex), 2 mM L-glutamine (Cambrex), 100 IU/mL penicillin-streptomycin, and 10000 IU/mL nystatin (antimycotic solution) (Cambrex) in a humidified atmosphere with 5% CO_2_ at 37°C. Culture medium was changed every 2 days.

The prooxidative stress was induced by incubating the fibroblasts with 1 mM H_2_O_2_ for 1 h at 37°C either alone or in combination with 1 or 2 mg/mL (w/v, in culture medium) of* C. papaya* seeds water extract or vitamin C (50, 100, 150, 200, and 250 *μ*M).* C. papaya *extract or vitamin C was added to the culture medium simultaneously with H_2_O_2_ and/or during the recovery (1 h) in fresh medium at 37°C.

### 2.4. Cell Viability and Morphology

*MTT Assay*. Cell viability was determined by 3-(4,5-dimethylthiazol-2-yl)-2,5-diphenyltetrazolium bromide (MTT) dye mitochondria reduction in living fibroblasts according to Sladowski et al. [18]. Briefly, 5 × 10^5^ fibroblasts were incubated with 1 mg/mL of MTT prepared in supplemented EMEM culture medium, for 2 h at 37°C and 5% CO_2_; fibroblasts were washed three times with 0.2 M Phosphate Buffer Saline (PBS) pH 7.4, and the reduced MTT formazan crystals were solubilised with DiMethyl SulfOxide (DMSO) (Carlo Erba, Milano, Italy). The optic density (OD) was read at the spectrophotometer (Ultrospec 4000 UV/Visible Spectrophotometer, Pharmacia Biotech, Stockholm, Sweden) at 570 nm.

*Light Microscopy*. Morphological changes of living fibroblasts were investigated with an inverted light microscope (LM) Eclipse TS100 (Nikon, Kawasaki, Kanagawa Prefecture, Japan). Fibroblasts, washed with 0.2 M PBS pH 7.4 and fixed with formalin 4% (v/v, in 0.2 M PBS pH 7.4), were rubber scraped and deposited on slides. Haematoxylin-eosin (H-E) staining slides were examined under a LM Eclipse 80i (Nikon) for the scoring of apoptosis, necrosis, or mitosis by counting at least 500 fibroblasts in 50–100 high-power microscopic fields (0.25 mm^2^). 

*Electron Microscopy*. Ultrastructure of cell was obtained by conventional transmission (TEM) and scanning electron microscopy (SEM). 10 × 10^6^ fibroblasts washed with 0.2 M PBS pH 7.4 and rubber scraped were fixed with 2.5% glutaraldehyde (v/v, in 0.1 M cacodylate buffer pH 7.4) for 1 h at ice temperature. After an extensive washing, fibroblasts were postfixed with 1% OsO_4_ (w/v, in 0.1 M cacodylate buffer pH 7.4) for 1 h at 4°C. Fibroblasts were dehydrated, embedded in Spurr's resin (TAAB Laboratories Equipment Ltd, Aldermaston, England), and examined under a CM12 TEM (Philips, Amsterdam, Netherlands) operating at 80 kV. An aliquot of 1% OsO_4_ postfixed fibroblasts was dehydrated by using a Critical Point Dryer 020 (Balzer, Balzers, Liechtenstein), stub-mounted, and gold-coated by using a Balzer Sputter Coater 040 before observation under a XL20 SEM (Philips) operating at 15 kV.

### 2.5. Catalase Activity

For catalase (EC 1.11.1.6.; 2H_2_O_2_ oxidoreductase) activity assay, fibroblasts were rinsed three times with 0.2 M PBS pH 7.4, rubber scraped, centrifuged at 450 g for 7–10 min at 4°C, and suspended in 1 mL of cold buffer (50 mM potassium phosphate pH 7, containing 1 mM EDTA). 3–5 × 10^6^ fibroblasts were sonicated for four cycles on ice (40% of amplitude, 10 s of sonication, and 5 s of pause) (Sonoplus Ultrasonic homogenizer HD 2070, Bandelin electronic, Berlin, Germany) and then centrifuged at 10000 g for 15 min at 4°C. Catalase activity was determined in the supernatants by using the FR20 kit following the manufacturer's instructions (Oxford Biomedical research, Oxford, USA).

### 2.6. Induction of Apoptosis

Fibroblasts were induced to apoptosis with 10 *μ*g/mL puromycin (PMC, in culture medium) for 6 h at 37°C in a 5% CO_2_ humidified atmosphere or with 10^−2^ M cycloheximide (CHX, in culture medium) for 24 h followed by recovery in fresh medium for an additional 1 h at 37°C in a 5% CO_2_ humidified atmosphere, in the absence or in the presence of 1 or 2 mg/mL (w/v, in culture medium) of* C. papaya *seeds water extract.

### 2.7. [Ca^2+^]

5 × 10^7^ fibroblasts washed twice with loading buffer (10^7^ mM NaCl, 5 mM KCl, 7 mM NaHCO_3_, 3 mM CaCl_2_, 1 mM MgSO_4_  ×  6H_2_O, 20 mM Hepes, 10 mM glucose, and 1% bovine serum albumin (BSA)) were incubated at the concentration of 10^6^ fibroblasts/mL with 4 mM 1-(2-(4-carboxyphenyl)-6-amino-benzofuran-5-oxy)-2-(2′-amino-5′-methylphenoxy)ethane-N,N,N′,N′-tetraacetic acid (Fura-2) acetoxymethyl ester for 45 min at 37°C in a 5% CO_2_ humidified atmosphere. After two washes with the loading buffer, fibroblasts were resuspended in the same freshly made buffer at a final concentration of 7 × 10^6^ fibroblasts/mL and stored at RT until use. 2 mL of cell suspension, prewarmed at 37°C for 20 min and adjusted at a final concentration of 1.4 × 10^5^ fibroblasts/mL, was placed in a glass cuvette for Fura-2 fluorescence measure with a FP-750 spectrofluorometer (Jasco Europe s.r.l., Lecco, Italy) equipped with an electronic stirring system and a thermostabilised (37°C) cuvette holder and monitored by a personal computer running the Jasco Spectra Manager software for Windows 95 (Jasco Europe s.r.l.). The excitation wavelengths were 340 and 380 nm and the emission wavelength was 510 nm; the slit widths were set to 10 nm. Fluorescence values were converted to [Ca^2+^]_*i*_ values according to Grynkiewicz et al. [19].

### 2.8. HSP-70

The inducible cytosolic Heat Shock Protein 70 (HSP-70) protein was detected by Western blot from the total cytosolic proteins of Detroit 550 fibroblasts. Proteins were separated by sodium dodecyl sulfate-polyacrylamide gel electrophoresis (SDS-PAGE) by using 12.5% acrylamide gels according to Laemmli [20] and then electrotransferred onto nitrocellulose paper sheets (Hybond-C extra, Amersham, UK) according to Towbin et al. [21]. Before electrophoresis, proteins (25 *μ*g) were denaturized with 2% SDS, 10% glycerol, 5% *β*-mercaptoethanol, and 0.05% bromophenol blue in 62.5 mM Tris-HCl (pH 6.8) for 4 min at 95°C. After blockage with 25 mM Tris-HCl (pH 8.0) containing 3% BSA, 127 mM NaCl, and 2.7 mM KCl (TBS buffer) and washing with TBS containing 0.05% Tween 20, nitrocellulose sheets were incubated with monoclonal anti-HSP-70 (1 *μ*g/mL) for 2 h at RT. Specific antibody binding was detected using goat anti-mouse immunoglobulin G (IgG) conjugated to biotin (1 *μ*g/mL), streptavidin conjugated to peroxidase (1 : 1,500 dilution), and 3-3′diaminobenzidine (DAB) as a substrate. Biotinylated SDS molecular weight (MW) standards were run in parallel to samples and stained. *β*-actin Western blots were used as controls. Quantification was performed by using a GS-700 Imaging densitometer (Bio-Rad, Hercules, CA, USA).

### 2.9. Mitochondrial Transmembrane Potential

Changes in the inner mitochondrial transmembrane potential (Δ*ψ*_*m*_) were determined by incubating fibroblasts with the fluorescent probe 5,5′,6,6′-tetrachloro-1,1′,3,3′-tetraethylbenzimidazolylcarbocyanine iodide (JC-1) (Cell Technology, JC-1 Kit, Mountain View, CA, USA) for 15 min at 37°C in the dark. JC-1 was dissolved in DMSO, stored, and used according to the manufacturer's instruction. The monomeric form of the lipophilic cation JC-1 remains in the cytoplasm and stains it in green, while the dimeric form enters the mitochondria with an intact membrane potential and stains them in red. The percentage of the organic solvent in the samples never exceeded 1% (v/v). Fibroblasts grown on coverslips were rapidly washed with 0.2 M PBS pH 7.4, drained to prevent buffer salt crystals formation, and air-dried. Once completely dried, coverslips were mounted with Histovitrex mounting medium (Carlo Erba, Milan, Italy) and observed with an epifluorescence LM Eclipse 80i (Nikon, Kawasaki, Kanagawa Prefecture, Japan) equipped with a C-HGFIE Hg precentered fiber illuminator (130 W) and a digital camera DXM 1200F by setting a FITC or TRITC filter.

### 2.10. Cytochrome C Quantification

Quantitative determination of cytosolic and mitochondrial cytochrome C was performed with an Assay Designs' human cytochrome C TiterZyme Enzyme Immunometric Assay (EIA) kit (Assay Designs Inc., Ann Arbor, MI, USA). Fibroblasts rinsed twice with 0.2 M PBS pH 7.4 were suspended in digitonin cell permeabilization buffer (250 nM sucrose, 137 mM NaCl, 70 mM KCl, 1.3 mM Na_2_HPO_4_, 1.4 mM K_2_HPO_4_, 0.2 mg/mL digitonin, and 0.1% hydorol M) to obtain a cytosolic fraction of cytochrome C and then in a radio-immunoprecipitation assay (RIPA) cell lysis buffer (50 mM Tris HCl pH 7.4, 150 mM NaCl, 1 mM EDTA, 1 mM EGTA, 1% Triton X-100, 1% sodium deoxycholate, and 0.1% SDS) to obtain a mitochondrial fraction of cytochrome C. Both fractions were incubated with a biotinylated monoclonal antibody to cytochrome C immobilized on a microtiter plate for 1 h. The excess of antibody was washed out and streptavidin conjugated to alkaline phosphatase was added for 30 min before the addition of pNPP (p-nitrophenyl phosphate) substrate for additional 45 min. The colour generated by the reaction was read at 405 nm, using the ETI-SYSTEM Fast Reader (Sorin Biomedica, Vicenza, Italy). The values were expressed as pg of cytochrome C of *μ*g total proteins. The protein concentration was measured by using a Bio-Rad Protein assay kit.

### 2.11. Statistical Analysis

Data were analysed by performing one-way analysis of variance (ANOVA) at the 95% confidence level. *P* values less than 0.05 were considered significant. Data are the means ± standard errors (SEs) of six independent experiments each done in duplicate.

## 3. Results

### 3.1. Cytotoxicity of* C. papaya* Seeds Water Extract

**Figure 2:**
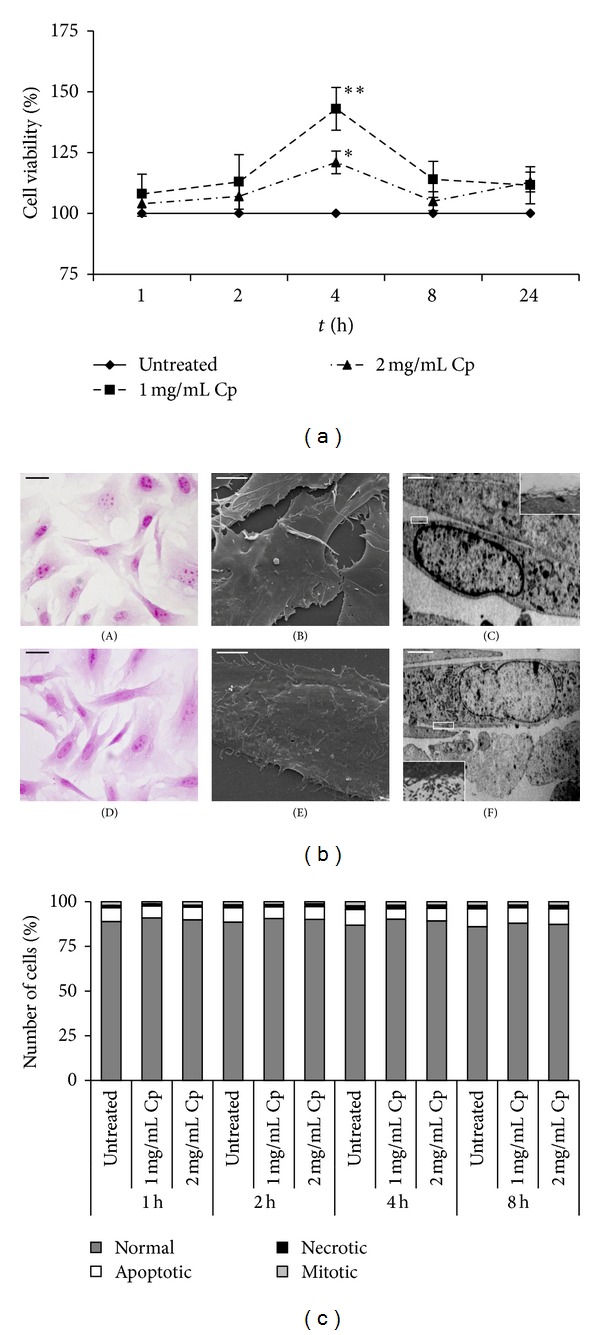
Viability and morphology of Detroit 550 fibroblasts treated with* C. papaya* seeds water extract. (a) Viability was assessed by MTT assay. Detroit 550 fibroblasts were treated with 1 or 2 mg/mL* C. papaya* (Cp) seeds water extract for 1, 2, 4, 8, and 24 hours (h). The values are reported as percentage of the control untreated fibroblasts considered as 100%. Each value represents the mean ± SE of six independent experiments, each done in duplicate. Single star indicates value significantly different from untreated control fibroblasts (*P* < 0.05). Two stars indicate value significantly different from the one star value (*P* < 0.05). (b) LM (A, D), SEM (B, E), and TEM (C, F) micrographs of Detroit 550 fibroblasts untreated (A–C) or treated with 1 mg/mL of* C. papaya* seeds water extract for 4 h (D–F). (A, D): H-E stained fibroblasts; bars = 20 *μ*m. (B, E): bars = 5 *μ*m. (C, F): the inset is a magnification showing microvilli; bars = 5 *μ*m. (c) Percentages of normal, apoptotic, necrotic, and mitotic Detroit 550 fibroblasts after treatment with 1 or 2 mg/mL* C. papaya* (Cp) seeds water extract for 1, 2, 4, and 8 h. At least 500 fibroblasts for each treatment were scored on H-E stained slides.

*C. papaya *seeds water extract was preliminary tested for cytotoxicity (Figure 2). The two concentrations of extract used in the present work (1 and 2 mg/mL) were never toxic at all incubation times. Indeed, 1 mg/mL extract increased cell viability of about 43% over the control value at 4 h of incubation (*P* < 0.05, Figure 2(a)). Likewise, H-E stained control (Figure 2(b), (A)) and 1 mg/mL extract 4 h treated Detroit 550 fibroblasts (Figure 2(b), (D)) exhibited the same spindle shape with a central large ovoid nucleus and many randomly distributed filopodia and microvilli that were better seen at SEM (Figure 2(b), (B)–(E)) and TEM (Figure 2(b), (C)–(F)) level. The TEM analysis allowed observing that these cytoplasmic protrusions were particularly abundant in fibroblasts incubated with 1 mg/mL of* C. papaya *seeds water extract (Figure 2(b), (C)–(F), insert). The percentages of apoptotic, necrotic, and mitotic cells were not modified between control* versus* treated fibroblasts during 8 h of culture (Figure 2(c)).

### 3.2. Antioxidant Activity of* C. papaya* Seeds Water Extract

**Figure 3:**
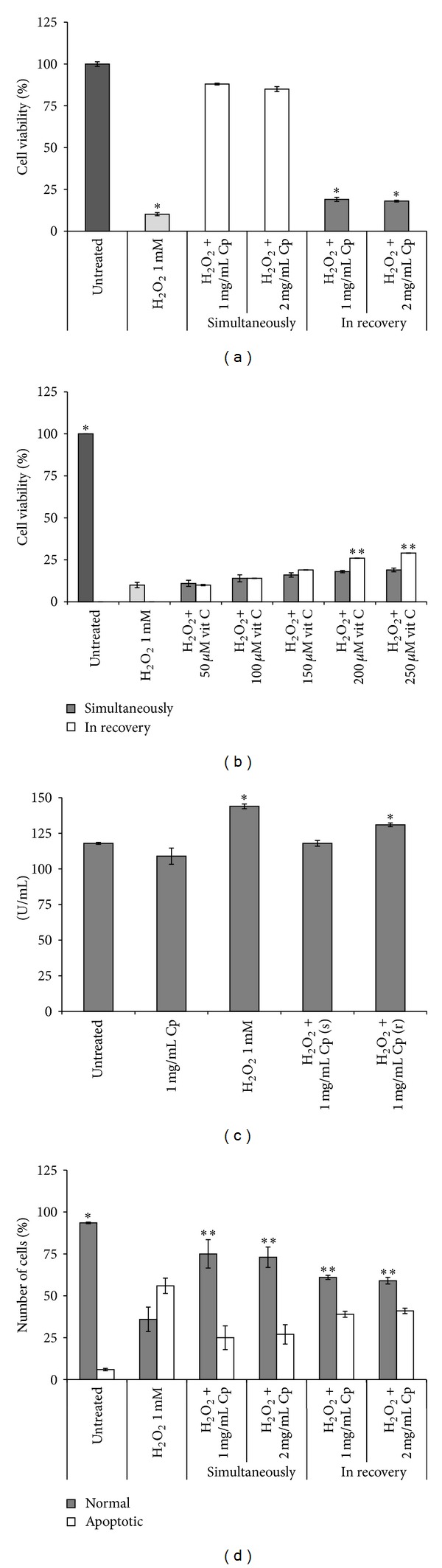
Antioxidant activity of* C. papaya* seeds water extract on Detroit 550 fibroblasts. (a-b) Viability was assessed by MTT assay. The values are reported as percentage of the control untreated fibroblasts considered as 100%. (a) Detroit 550 fibroblasts were treated with 1 or 2 mg/mL* C. papaya* (Cp) seeds water extract simultaneously with 1 mM H_2_O_2_ for 1 h or during the recovery. Single star indicates values significantly different from untreated control fibroblasts (*P* < 0.05). (b) Detroit 550 fibroblasts were treated with different concentrations of vitamin C (vit C) simultaneously with 1 mM H_2_O_2_ for 1 h or during the recovery. Single star indicates that the value is significantly different from all others (*P* < 0.05). Two stars indicate values significantly different from those marked with one star and from the simultaneous treatment value (*P* < 0.05). (c) Catalase enzyme activity (U/mL) measured in Detroit 550 fibroblasts treated with 1 mg/mL of Cp seeds water extract added simultaneously (s) with 1 mM H_2_O_2_ for 1 h or during recovery (r). Single star shows values significantly different from all others (*P* < 0.05). (d) Percentages of normal and apoptotic Detroit 550 fibroblasts after treatment with 1 mM H_2_O_2_ administrated either alone or in combination with 1 or 2 mg/mL of Cp extract or with Cp extract added during 1 h recovery. At least 500 fibroblasts for each treatment were scored on H-E stained slides. Single star shows a value significantly different from all others and from the corresponding apoptotic untreated control fibroblasts (*P* < 0.05). Two stars indicate values significantly different from the untreated control fibroblasts and from the corresponding apoptotic ones (*P* < 0.05). Each value represents the mean ± SE of six independent experiments, each done in duplicate.

The antioxidant activity of* C. papaya *seeds water extract was assayed on fibroblasts in which H_2_O_2_-mediated oxidative stress was induced.* C. papaya *seeds water extract protected fibroblasts from the oxidative stress only when added simultaneously with H_2_O_2_ (Figure 3(a)). In fact, cell viability was increased on extract-treated fibroblasts with respect to the fibroblasts incubated only with H_2_O_2_, thus suggesting an antioxidant activity. However, the extract was unable to protect fibroblasts when added during the recovery (Figure 3(a)). No differences in the antioxidant efficacy were found between the two extract concentrations.

The antioxidant activity of* C. papaya *seeds water extract was compared with a well-known antioxidant, vitamin C (Figure 3(b)). No toxic concentrations of vitamin C (50, 100, 150, 200, and 250 *μ*M) were added to the culture medium simultaneously with H_2_O_2_ or during recovery. Antioxidant vitamin C effect was differently exerted than extract. In fact, vitamin C was more efficient to hamper the oxidative stress when added after oxidative stress ignition than simultaneously with H_2_O_2_ in a concentration dependent manner (Figure 3(b)). Interestingly,* C. papaya *seeds water extract was, in absolute, more efficient than vitamin C to protect Detroit 550 fibroblasts against the oxidative stress. In fact, cell viability increased about 9 times with 1 mg/mL* C. papaya *seeds water extract (added during 1 h H_2_O_2_ incubation) and about 3 times with the most efficient concentration of vitamin C (200 *μ*M added during recovery) with respect to H_2_O_2_ treatment alone (please compare Figures 3(a) and 3(b)).

### 3.3. Catalase Activity

Catalase enzyme is one of the most efficient antioxidant molecules of eukaryotic living cells by hydrolysing H_2_O_2_ to water and oxygen. The H_2_O_2_-induced oxidative stress increased catalase activity of about 1.2-fold over the control (*P* < 0.05, Figure 3(c)). When the extract was added simultaneously to the oxidative stress, catalase enzyme activity was similar to the control value (Figure 3(c)). The reduction of the catalase activity was only partially observed (−10% than H_2_O_2_ treatment alone) when the extract was added during the recovery (*P* < 0.05, Figure 3(c)).

### 3.4. Antiapoptotic Activity of* C. papaya* Seeds Water Extract

**Figure 4:**
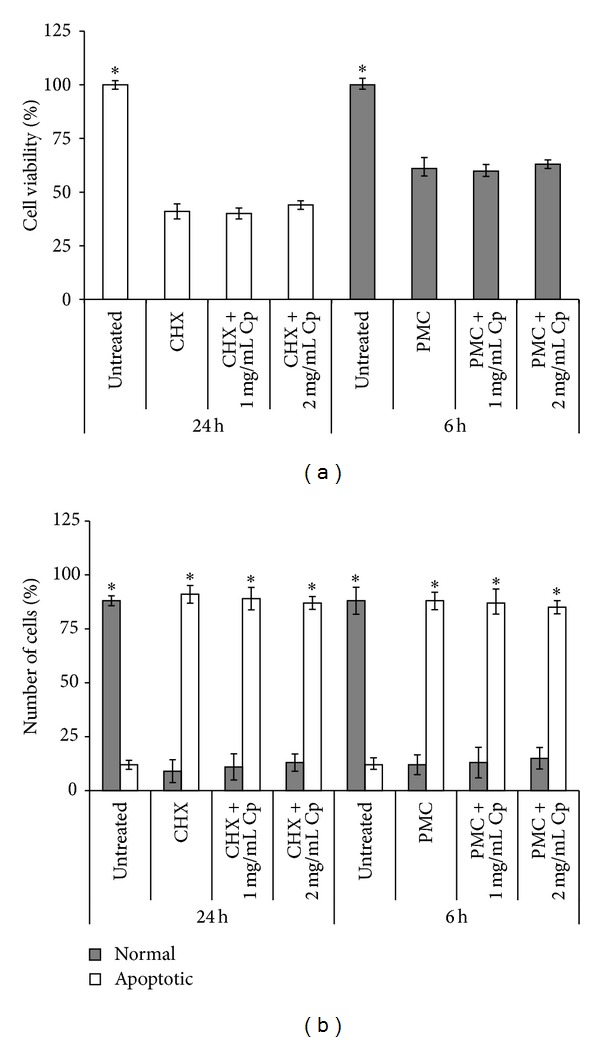
Antiapoptotic activity of* C. papaya* seeds water extract on Detroit 550 fibroblasts. Detroit 550 fibroblasts were treated with 10^−2^ M cycloheximide (CHX, in culture medium) or 10 *μ*g/mL puromycin (PMC, in culture medium) either alone or in combination with 1 or 2 mg/mL of* C. papaya* (Cp) extract for 24 or 6 hours (h), respectively. (a) Viability was assessed by MTT assay. Stars show significant value with the corresponding 24 or 6 h values (*P* < 0.05). (b) Percentages of normal and apoptotic fibroblasts. At least 500 fibroblasts for each treatment were scored on H-E stained slides. Single star indicates values that differ significantly from the corresponding normal or apoptotic ones (*P* < 0.05). Each value represents the mean ± SE of six independent experiments, each done in duplicate.

H_2_O_2_ represents also an inducer of apoptosis through the production of reactive oxygen species (ROS) [22]; thus the ability of the* C. papaya *seeds water extract to reduce the percentage of apoptotic fibroblasts was assayed. Data are reported in Figure 3(d). Both concentrations of* C. papaya *seeds water extract very efficiently decreased the number of apoptotic fibroblasts of about 30% when added during the H_2_O_2_-mediated oxidative stress (*P* < 0.05, Figure 3(d)). In fact, cell viability was comparable to untreated ones (Figure 3(a)). Conversely, any antiapoptotic effect of both concentrations of* C. papaya *seeds water extract was observed when added during recovery, as indicated by the unchanged viability (Figure 3(a)) and apoptotic cells values (Figure 3(b))* versus* H_2_O_2_ treatment alone. To verify if* C. papaya *seeds water extract exerts protection from apoptosis induced through pathways different from oxidative stress, fibroblasts were incubated with CHX and PMC that are able to induce apoptosis by inhibiting protein synthesis. Data of Figure 4 show that induction of apoptosis was unaffected by the presence of* C. papaya *seeds water extract added simultaneously to CHX or PMC treatments (Figures 4(a) and 4(b)). Comparable results were obtained by biochemical and morphological analysis.

Altogether these data suggest that inhibition of H_2_O_2_-mediated apoptosis is not exerted on the apoptotic process itself but it is the result of the antioxidant activity of* C. papaya *seeds water extract. In fact, no inhibition of H_2_O_2_-mediated apoptosis was observed when the extract was added while oxidative stress is already ignited.

### 3.5. Mitochondrial Δ*ψ*_*m*_ and Cytochrome C

**Figure 5:**
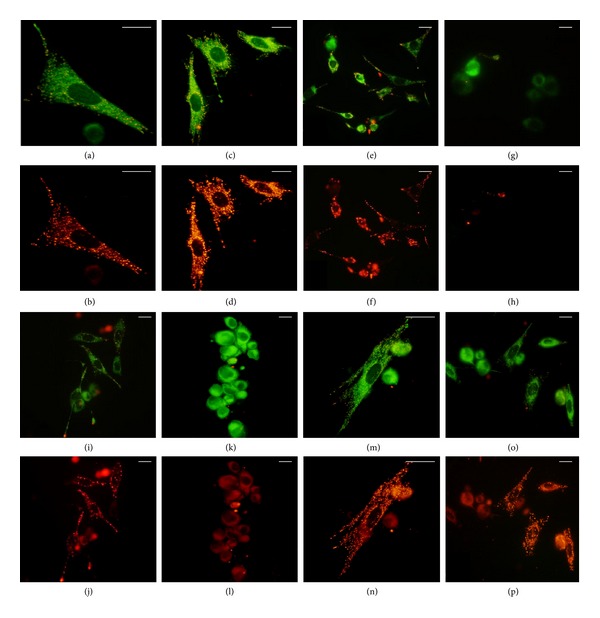
Detroit 550 fibroblasts labelled with the fluorescent probe JC-1 after the treatment with the* C. papaya* seeds water extract and/or with H_2_O_2_. Fibroblasts were labelled with JC-1 (1% v/v) and green or red fluorescence was observed with an epifluorescence LM by setting a FITC (a, c, e, g, i, k, m, and o) or TRITC filter (b, d, f, h, j, l, n, and p). (a)–(d): control untreated fibroblasts at T0 (a, b) and at 4 h (c-d); (e)-(f): fibroblasts treated with 1 mg/mL of* C. papaya* (Cp) extract for 4 h; (g)-(h): fibroblasts incubated with H_2_O_2_ 1 mM for 1 h; ((i)-(j)): fibroblasts incubated with 1 mM H_2_O_2_ and 1 mg/mL of Cp simultaneously for 1 h; (k)-(l): fibroblasts incubated with 1 mM H_2_O_2_ for 1 h and with 1 mg/mL of Cp extract added during recovery; (m)-(n): cell treated with 1 mg/mL of the subfraction with MW > 10 kDa (see Figure 1); (o)-(p): fibroblasts simultaneously incubated with 1 mg/mL of the subfraction with MW > 10 kDa and 1 mM H_2_O_2_ for 1 h. Bars = 20 *μ*m.

**Figure 6:**
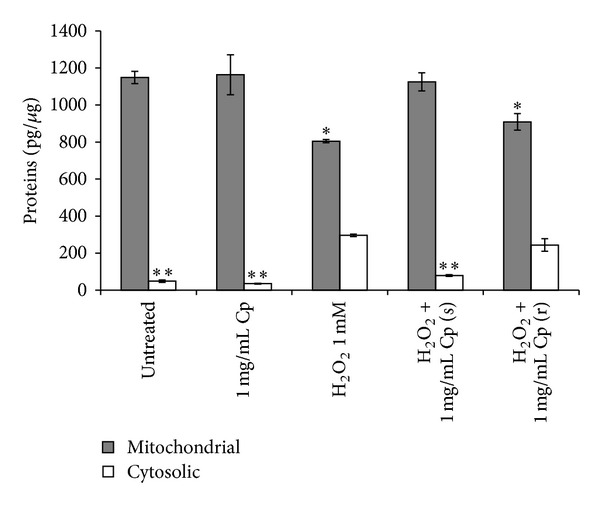
Cytoplasmic and mitochondrial cytochrome C levels. Detroit 550 fibroblasts incubated with 1 mM H_2_O_2_ for 1 h simultaneously (s) or during recovery (r) with 1 mg/mL of* C. papaya* (Cp) seeds water extract were harvested for the content of mitochondrial and cytosolic cytochrome C by ELISA assay. Concentrations are expressed as pg/*μ*g of total proteins. Single star indicates a significant value* versus* the untreated control fibroblasts (*P* < 0.05); two stars indicate a significant value* versus* all the others values relative to the cytosolic cytochrome C (*P* < 0.05). Each value represents the mean ± SE of six independent experiments, each done in duplicate.

Oxidative stress induces the loss of Δ*ψ*_*m*_ and the release of cytochrome C into the cytoplasm. These two events trigger the onset of apoptosis* via* the intrinsic pathway. The Δ*ψ*_*m*_ was evaluated by the high sensitive fluorescent probe JC-1. The monomeric form of JC-1 labels the cytoplasm of fibroblasts in brilliant green, while the dimeric form can enter into mitochondria with a normal Δ*ψ*_*m*_ and stain them in red. Data are shown in Figures 5 and 6. JC-1 labelled fibroblasts treated with 1 mg/mL of* C. papaya *seeds water extract did not alter Δ*ψ*_*m*_ (Figures 5(e) and 5(f)), while 1 mM of H_2_O_2_ induced the loss of Δ*ψ*_*m*_, thus preventing the passage of JC-1 dimers into the mitochondria (Figures 5(g) and 5(h)). When 1 mg/mL of* C. papaya *seeds water extract was added simultaneously to the oxidative stress, it prevented the Δ*ψ*_*m*_ loss and, thus, mitochondria were red stained (Figures 5(i) and 5(j)). The extract was unable to hamper Δ*ψ*_*m*_ loss when added during recovery (Figures 5(k) and 5(l)).

Mitochondrial Δ*ψ*_*m*_ loss causes in turn changes in the permeability of the outer mitochondrial membrane and provokes the release of cytochrome C, a well-known apoptotic-inducing factor. The cytosolic concentration of cytochrome C increased 6-fold than the control value during oxidative stress, that is, 300 pg* versus* 50 pg, respectively (*P* < 0.05, Figure 6). The addition during the oxidative stress of 1 mg/mL* C. papaya *seeds water extract prevented the release of cytochrome C. No effect has been observed upon the administration of the extract after the oxidative stress induction, as indicated by the unchanged cytochrome C concentration values with respect to H_2_O_2_ damaged fibroblasts (Figure 6).

### 3.6. [Ca^2+^] Ions

**Figure 7:**
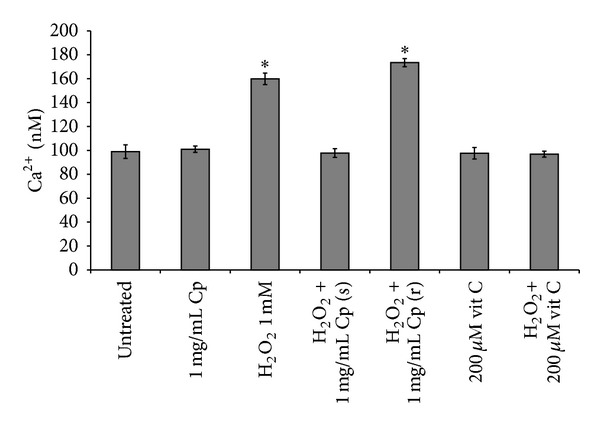
Ca^2+^ ions. Ca^2+^ ions concentration (nM) found after incubation of Detroit 550 fibroblasts with 1 mg/mL* C. papaya* (Cp) seeds water extract or with 200 *μ*M vitamin C (vit C) added simultaneously (s) or during recovery (r) with 1 mM H_2_O_2_ either for 1 h. The values indicated with the single star differ significantly from all the others (*P* < 0.05). Each value represents the mean ± SE of six independent experiments, each done in duplicate.

Oxidative stress induces Ca^2+^ ions influx into the cytoplasm, which in turn causes disruption of the normal physiological pathways and leads to cell death. Ca^2+^ ions influx into the cytoplasm was prevented by 1 mg/mL of* C. papaya *seeds water extract when added simultaneously to H_2_O_2_ (Figure 7). The same behaviour was observed with addition of 200 *μ*M vitamin C either alone or simultaneously with 1 mM H_2_O_2_ added for 1 h. [Ca^2+^] ions concentration was 160 nM in H_2_O_2_ treated fibroblasts and 100 nM in control and extract or vitamin C treated ones (*P* < 0.05, Figure 7).

### 3.7. HSP-70

**Figure 8:**
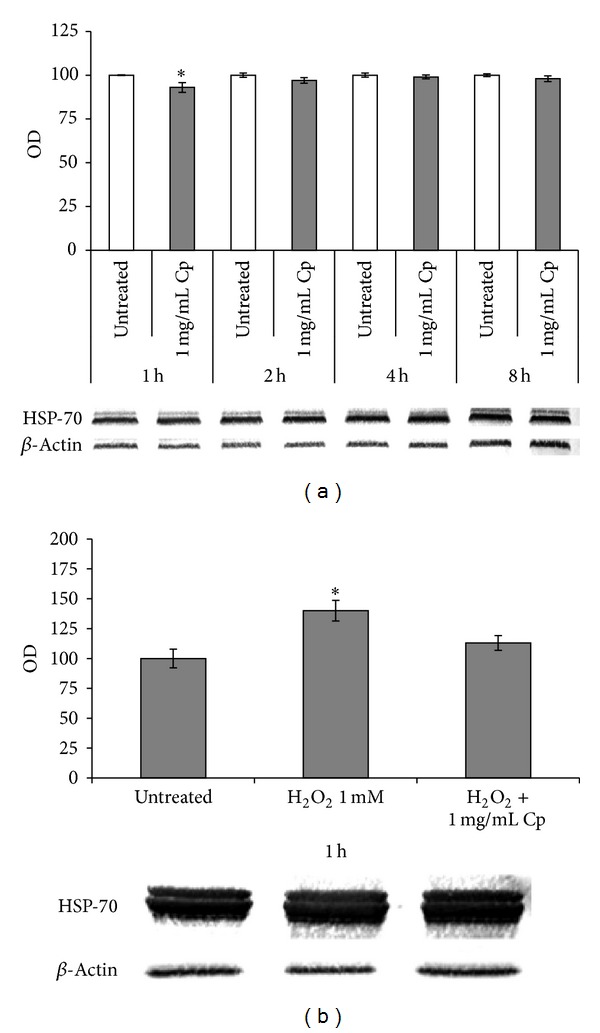
Western blot of HSP-70. (a) Western blots and optic densities (OD) of inducible cytosol HSP-70 of control untreated Detroit 550 fibroblasts or treated with 1 mg/mL of* C. papaya* (Cp) seeds water extract for 1, 2, 4, and 8 hours (h); (b) Western blots and OD of inducible cytosol HSP-70 of Detroit 550 fibroblasts incubated with 1 mM H_2_O_2_ for 1 h in absence or in presence of 1 mg/mL of Cp seeds water extract. Values are expressed as percentage of the control untreated fibroblasts value taken as 100%. The star shows a significant value with respect to all the others without star (*P* < 0.05). Each value represents the mean ± SE of six independent experiments, each one done in duplicate.

HSPs protect cells against a plethora of stresses, including oxidative stress. A reduction of 7% of the amount of HSP-70 was observed when fibroblasts were treated for 1 h with 1 mg/mL* C. papaya* seeds water extract (*P* < 0.05, Figure 8(a)). Overexpression of HSP-70 protein was found upon 1 mM H_2_O_2_ incubation of Detroit 550 fibroblasts (*P* < 0.05, Figure 8(b)).* C. papaya *seeds water extract, added during the oxidative stress but not during the recovery, hampered the stress by decreasing the amount of HSP-70 to control value (Figure 8(b)).

### 3.8. Antioxidant Activity of* C. papaya* Seeds Water Extract Subfractions

**Figure 9:**
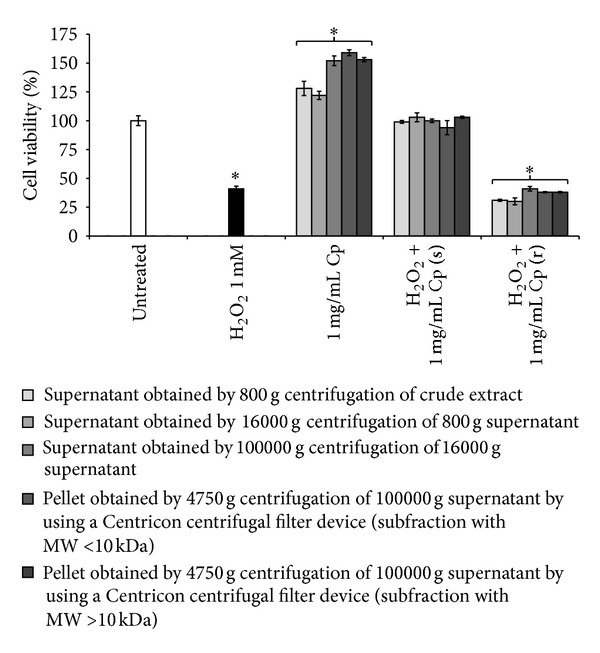
Viability of Detroit 550 fibroblasts treated with different subfractions of* C. papaya* seeds water extract. Viability of Detroit 550 fibroblasts treated with 1 mg/mL (w/v, in the culture medium) of different subfractions obtained from the* C. papaya* (Cp) extract (see Figure 1) administrated either alone or with 1 mM H_2_O_2_ added simultaneously (s) or during 1 h recovery (r) was evaluated by MTT test. One star indicates values significantly different from control untreated fibroblasts (*P* < 0.05). Each value represents the mean ± SE of six independent experiments done in duplicate. For the preparation of subfractions, see Figure 1 and Materials and Methods section.

The fractionation of* C. papaya *seeds water extract by differential centrifugation (Figure 1) allowed us to evaluate if the antioxidant effect of extract was an exclusive property of the whole crude extract or was mainly related to a specific class of compounds. Any of the different subfractions (1 mg/mL final concentration) was cytotoxic as shown with MTT assay and JC1 staining (Figures 9 and 5, resp.).  Figure 5 shows red stained mitochondria of Detroit 550 fibroblasts treated with MW > 10 kDa subfraction alone (Figures 5 (m) and 5(n)) and simultaneously with H_2_O_2_ (Figures 5(o) and 5(p)). The subfractions increased viability of Detroit 550 fibroblasts: the maximum increment was measured with the supernatant obtained by 100000 g centrifugation of 16000 g supernatant (*P* < 0.05, Figure 9), especially with the subfraction containing substances with MW < 10 kDa (Figure 9). Regarding the antioxidant activity, the different subfractions were not significantly different than the whole extract when added simultaneously with the oxidative stress (Figure 9). Any of the subfractions could reduce the oxidative stress when added during recovery.

## 4. Discussion

The present study shows that* C. papaya *seeds water extract has a potent antioxidant activity in H_2_O_2_ oxidative stress-induced human skin Detroit 550 fibroblasts. Our results suggest that the extract is not toxic, decreases cell death, ensures Ca^2+^ homeostasis, and counteracts mitochondrial dysfunctionality in oxidative stress-damaged Detroit 550 fibroblasts. The best effect primarily depends on the modality of administration and to a lesser extent on the dose and composition, that is, whole extract* versus* subfractions. We found that (i) the protective effect was exerted when the extract was added concomitantly to the oxidative stress, suggesting that the bioactive compounds of* C. papaya* extract are not inactivated by H_2_O_2_ (as it is for vitamin C) but, conversely, can scavenge H_2_O_2_ but not free radical; (ii) the sufficient dose to induce antioxidant effect in our experimental design is 1 mg/mL, likely due to the concentration of the diverse bioactive molecules; (iii) the crude whole extract is effective as much as the subfractions.

Thus, our data strongly highlight the antioxidant role of the molecules contained in* C. papaya* seeds water extract, encouraging their exploitation. In fact, substances with antioxidant properties have recently received unprecedented attention as possible therapeutic and preventive agents in consideration of the role of ROS in health and diseases. In addition, due to the augmented recycling interest of the agrofood industry, the numbers of studies on residual sources in replacing synthetic antioxidants with natural ones are increasing. The wastes or byproducts from food processing such as seeds and peels contain higher source of potential antioxidant compounds than the edible portion [23]; conversely, byproduct-derived antioxidants successfully developed are very limited. Grape seed and olive waste extract in the European food processing industry represent these exceptions [24]. In this context,* C. papaya *seeds water extract could be a source to exploit. In fact, papaya seeds contain several molecules, like fatty acids, crude protein, crude fiber, papaya oil, carpaine, benzyl isothiocyanate, glucotropaeolin, benzyl thiourea, hentriacontane, *β*-sitosterol, caricin, and an enzyme myrosin [7] that confer vermifuge [25, 26], abortifacient [27, 28], antifertility [29–32], wound-healing [33], anticancer [34], antibacterial [35], and antifungal [36] properties as well as antioxidant activities for the presence of secondary metabolites, such as phenolic compounds, vanillic acid, flavonoids, *α*-tocopherol, and vitamin C. Despite the wide and historical use of* C. papaya *in the traditional management of many diseases [4], the scientific validation of the use of papaya seeds as antioxidant is scarce. Therefore, in the present work we tested* C. papaya *seeds water extract that conforms to the use in folk medicine and ensures no toxicity related to chemicals used during the preparation. In our hands,* C. papaya *seeds water extract is not toxic and, as a whole, it is more efficient than vitamin C to hamper the H_2_O_2_ oxidative damage.

The choice of the methodology for extract preparation is very important to preserve antioxidant activity [17, 37]. The antioxidant activity of seed extract could be further improved, taking into account that the comparison of five extract fractions, that is, ethanol, petroleum ether, ethyl acetate, n-butanol, and water, demonstrated the strongest antioxidant activity of the ethyl acetate and n-butanol fractions, whereas the water extract had always the weakest antioxidant activity [17]. Despite this data, we tested the water extract because it is edible, thus compatible with a putative human administration. Here, we also demonstrated that the modality of administration is crucial as well. Since experimental H_2_O_2_ oxidative stress is based on two-step procedure, that is, 1 mM H_2_O_2_ for 1 h followed by 1 h of recovery in fresh medium, we found that the best modality of papaya extract administration is when it is added simultaneously with the oxidative stress inducer. This suggests that the extract acts directly with H_2_O_2_. In addition,* C. papaya* seeds water extract was, as a whole, much more efficient than vitamin C, a well-known antioxidant agent. In fact, vitamin C had its highest antioxidant activity when added in the recovery. H_2_O_2_ must be firstly converted by fibroblasts to free radicals that can be then neutralized by vitamin C. In fact, ascorbic acid or vitamin C is a water soluble antioxidant that delays or inhibits cellular damage mainly through free radical scavenging. Vitamin C belongs to primary antioxidants group that are able to donate electrons to free radical, scavenging them to compounds more stable and not harmful to cells [38].

The robust antioxidant activity of* C. papaya* seed extract can protect from cell death. In fact, we showed the ability of the extract to prevent H_2_O_2_-induced apoptosis. However, the extract does not affect directly the apoptotic process but indirectly by hampering the oxidative stress-induced apoptosis. In fact,* C. papaya* extract has no efficacy against CHX- and PMC-induced apoptosis, inhibitors of protein synthesis. Therefore, the protection from the oxidative-stress-induced apoptosis is an indirect consequence of the ability of the extract to prevent the production or to block the activity of harmful metabolites, like free radicals.

Antioxidant function in biological systems is much more complicated than a simple free radical scavenging process. An antioxidant may affect biological system by (i) suppressing the formation of ROS and reactive nitrogen species (RNS); (ii) affecting enzyme activities [39]; (iii) inducing* de novo* biosynthesis of defence enzymes and thereby affecting other endogenous antioxidants [40]; (iv) preserving NO activity [41], or (v) sequestering transition metal ions. It is obvious that some compounds use more than one mechanism for their antioxidants effect on biological systems. Our data show that the antioxidant effect of* C. papaya *seeds water extract did not increase scavenging enzymes activity, as catalase. When added simultaneously to the oxidative stress, the extract reduces the catalase activity to normal level. This suggests that the antioxidant effects of the* C. papaya* seeds water extract are exerted not* via* modulation of catalase enzyme activity but by (i) scavenging and chelating the oxidative molecules (H_2_O_2_) or (ii) reestablishing or maintaining the reduction/oxidation balance inside the cell.

Oxidative stress causes mitochondrial dysfunction* via* loss of Δ*ψ*_*m*_, accumulation of mitochondrial DNA mutations, and increment of the level of oxidative damage to DNA, proteins, and lipids [1]. Yakes and Van Houten [42] demonstrated that H_2_O_2_ treatment affects mainly mitochondria rather than DNA in human cells. In our system,* C. papaya *seeds water extract prevents the Δ*ψ*_*m*_ loss and the consequent release of cytochrome C into the cytosol.

Cytochrome C is normally bound to the inner mitochondrial membrane by association with cardiolipin, whose peroxidation leads to cytochrome C and release through the outer mitochondrial membrane into the cytosol. Once in the cytoplasm, cytochrome C triggers the activation of caspase-9 that activates the intrinsic pathway of apoptosis [43]. Since* C. papaya *seeds water extract prevents oxidative-induced cell death by blocking the cytochrome C release from mitochondria to cytosol, by reducing the mitochondrial ROS and by inhibiting the mitochondrial permeability transition, we speculate that it is cell permeable and able to target mitochondria.

Oxidative stress burst can disrupt normal physiological pathways that, in turn, trigger the Ca^2+^ signaling dependent cell death. In fact, oxidative stress causes Ca^2+^ influx into the cytoplasm from the extracellular environment and from the endoplasmic reticulum (ER) through, respectively, cell membrane and ER channels [44].* C. papaya *seeds water extract elicits antioxidant effect also by preventing the H_2_O_2_-induced alteration of Ca^2+^ homeostasis. The significant cytosolic HSP-70 induction occurred after exposure to H_2_O_2_ was decreased by* C. papaya *seeds water extract to normal level. These results point to the possible involvement of redox mechanisms in the heat shock signal transduction pathway, which may play an important regulatory role in the genetic mechanisms of tolerance to oxidative stress.

The antioxidant properties of five subfractions of the water crude extract obtained by differential centrifugation were tested in the same conditions of the whole extract. Each subfraction showed activities comparable to those tested for the whole extract, thus indicating that the antioxidant activity of the* C. papaya* seeds water extract is not entirely attributable to only one molecule or few association of molecules and that there is no synergistic activity of the bioactive compounds. It is worth noting that one of the five subfractions tested expressed a property that was not shown by the other subfractions. In fact, the subfraction with MW > 10 kDa was able to counteract the loss of Δ*ψ*_*m*_. The reason of such behavior needs further investigations.

Altogether our data are in favour of a beneficial effect of the exogenous supplementation of* C. papaya *seeds water extract (as whole or as MW > 10 kDa subfraction) representing an efficient tool to minimize free radical-induced damage.

## 5. Conclusions

In conclusion, this study indicates that also nonedible parts of* C. papaya*, in particular seeds water extract, may be a promising source of antioxidants, which may have therapeutic implications. In fact, we showed that* C. papaya *seeds protect fibroblasts from H_2_O_2_-induced stress due to the antioxidant activity of the water extract. All together our results gave indication on the benefits, on the modality of administration, and on the extraction of* C. papaya *seeds that are important for the extent of the antioxidant activity and open a new perspective in the biotechnological utilization of the* C. papaya*. Indeed, this aspect could be very important to recycle the waste of papaya fruit, playing an important role to improve the complete utilization of its nonedible byproducts.
